# The impact of a high-protein diet with strength training on the gastrointestinal microbiota in community-dwelling older adults: subanalysis of a randomized controlled trial

**DOI:** 10.3389/fnut.2025.1712451

**Published:** 2026-01-20

**Authors:** Patrick A. Zöhrer, Sandra Unterberger, Rudolf Aschauer, Agnes Draxler, Sophie Somloi, Maria Kapeller, Teresa Bauer, Cornelia Heinz, Stefanie Reichstam, Bernhard Franzke, Eva-Maria Strasser, Bela Hausmann, Petra Pjevac, David Berry, Barbara Wessner, Karl-Heinz Wagner

**Affiliations:** 1 Research Platform Active Ageing, University of Vienna, Vienna, Austria; 2 Department of Nutritional Sciences, Faculty of Life Sciences, University of Vienna, Vienna, Austria; 3 Vienna Doctoral School of Pharmaceutical, Nutritional and Sport Sciences (PhaNuSpo), University of Vienna, Vienna, Austria; 4 Centre for Sport Science and University Sports, University of Vienna, Vienna, Austria; 5 Research Center Health Sciences, University of Applied Sciences Campus Vienna, Vienna, Austria; 6 Karl Landsteiner Institute for Remobilization and Functional Health/Institute for Physical Medicine and Rehabilitation, Kaiser Franz Joseph Hospital, Social Medical Center South, Vienna, Austria; 7 Joint Microbiome Facility, Medical University of Vienna and University of Vienna, Vienna, Austria; 8 Department of Laboratory Medicine, Division of Clinical Microbiology, Medical University of Vienna, Vienna, Austria; 9 Centre for Microbiology and Environmental Systems Science, Department of Microbiology and Ecosystem Science, Division of Microbial Ecology, University of Vienna, Vienna, Austria

**Keywords:** gastrointestinal microbiota, microbiota diversity, high-protein diet, older adults, strength training, food intervention

## Abstract

**Background:**

A balanced gastrointestinal (GI) microbiota is essential for healthy aging. Although high-protein diets and strength training are recommended for older adults to maintain muscle mass, their effects on GI microbiota remain unclear.

**Methods:**

This randomized controlled trial examined the effect of a habitual diet with recommended protein intake or high protein intake combined with strength training on the GI microbiota of 112 community-dwelling adults aged 65–85 years. The participants were divided into three groups: no intervention control (CON), recommended protein intake plus strength training (RP + T), and high protein intake plus strength training (HP + T). Over 17 weeks, protein intake increased significantly from 0.80 (IQR: 0.30–0.50) g/kg body weight at baseline, reaching 1.07 ± 0.25 g/kg in RP + T, and 1.62 ± 0.37 g/kg in HP + T groups. Stool samples collected at baseline, after dietary intervention, and after combined dietary and training intervention were analyzed using 16S rRNA gene amplicon sequencing.

**Results:**

Despite increased protein intake, microbiota richness, diversity, and composition showed no significant changes within or between groups. Residual energy and inflammatory markers indicated that higher protein intake was well tolerated.

**Conclusion:**

The findings suggest that increasing protein intake via food sources up to 1.6 g/kg body weight for more than 4 months, with or without strength training, does not adversely affect the GI microbiota composition in older adults.

## 1 Introduction

The population is steadily aging, with the number of people aged 80 years and older projected to triple by 2050 (1, 2). This increase in lifespan is associated with an increased risk of chronic diseases, such as type 2 diabetes, cancer, cardiovascular diseases, and dementia, as well as loss of muscle mass and independence (3). To preserve or enhance muscle mass in older adults, a combination of adequate protein intake and regular resistance training is recommended and has been substantiated in various studies (4).

Another important age-related modification involves the gastrointestinal (GI) microbiome, which is characterized by reduced microbiota diversity with increasing age. Age-related changes include a reduced abundance of beneficial bacteria such as *Bifidobacterium* and *Lactobacillus*, and the beneficial metabolites they produce, together with an increase in opportunistic pathogens and microorganisms related to chronic inflammation that might be associated with age-related chronic diseases (5, 6).

However, the link between high-protein diets and potential changes in GI microbiota in older adults has been barely investigated. Proteins have received increasing attention due to their role as key precursors in the formation of physiologically beneficial short-chain fatty acids (SCFAs) but also harmful putrefactive metabolites (such as ammonia, amines, sulfides, phenols, and indoles). The latter can be produced by the GI microbiota through proteolytic fermentation and may influence host health and contribute to the risk of diseases (7). Undigested proteins that reach the large intestine may lead to an increased abundance of opportunistic pathogenic microorganisms, potentially increasing the risk of metabolic diseases (8). Therefore, it is important to investigate whether the potential benefit of a high-protein diet on muscle mass or muscle quality might counteract GI health in older adults, particularly when the diet is provided via protein-rich foods rather than supplements, which might also change diet quality and composition. In an initial study involving 28 healthy elderly men who consumed a protein-rich diet—either at the level of the Recommended Daily Allowance (RDA) or twice that amount—for a duration of 10 weeks, no significant differences were observed in the composition of fecal microbiota or in the abundance of protein-associated volatile organic compounds. However, circulating trimethylamine-N-oxide (TMAO), a bacterial metabolite derived from choline and carnitine, and a potential novel biomarker for CVD, was increased by the 2x RDA diet (9).

Since no more data in humans are available, the goal of this randomized controlled trial was to compare the influence of a habitual diet with either a diet containing the recommended protein intake or a much higher protein intake, both with and without strength training, on the GI microbiota (as a secondary outcome analysis) in more than 110 older adults aged 65–85.

## 2 Materials and methods

### 2.1 Study design

This study was designed as a randomized controlled, observed-blind trial. After participants completed baseline assessments, they were allocated to one of three groups based on planned protein intake during the nutritional intervention (CON, control with no intervention; RP + T, recommended protein intake; HP + T, respectively, elevated high protein intake) as randomly permuted blocks via https://www.randomizer.at/ stratified by age groups [(65–70), (70–75), (75–80), (80–85) years] and sex (female; male) meeting the criteria for an observed-blind trial (10). The “+T”-groups additionally performed resistance training during the intervention. This resulted in eight different strata, and for every stratum block, randomization was performed. The randomization scheme was maintained by an independent researcher who was not involved in participant recruitment or baseline assessments, ensuring that allocation concealment was preserved throughout the enrollment process. A detailed description of the study with inclusion/exclusion criteria has been published previously (11).

This study evaluated the outcomes of the interventions on GI microbiota composition of stool samples collected during the study at three time points between July and December 2018 at the Center for Sport Science and University Sports, University of Vienna, Austria.

The study was approved by the Ethics Committee of the University of Vienna (Reference Number: 00322), registered at https://clinicaltrials.gov (NCT04023513), and performed in accordance with the 1975 Declaration of Helsinki.

### 2.2 Participant selection

The study population was defined as community-dwelling people aged between 65 and 85 years.

Regular resistance training in the past 6 months prior to the intervention was set as an exclusion criterion, as well as cognitive impairment evaluated by the Mini-Mental State Examination with a Score below 23 points, a frailty index ≥3, need for walking aids, and acute or chronic diseases contraindicating resistance training (11). None of the study participants reported antibiotic use at the time of recruitment; antibiotic use during the 17-week intervention was monitored.

**Figure 1:**
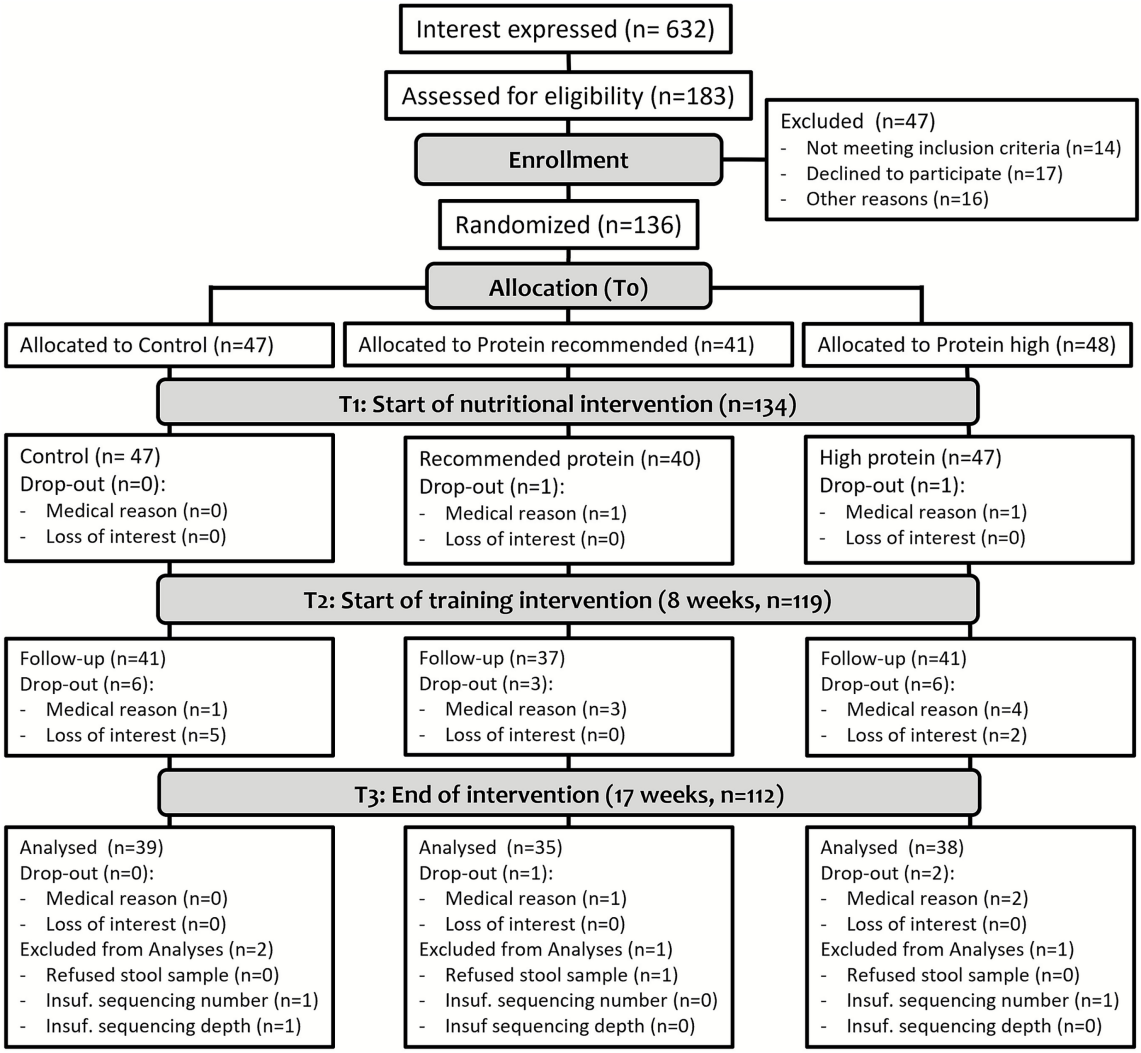
Flowchart of the study, adapted according to the dropouts in this microbiota-focused part (11).

A total of 137 of the 632 screened participants met the inclusion criteria and signed a written consent form before participation and allocation to the study groups (see Table 1 and Figure 1). Participants were only included in the final microbiota analysis if they met all inclusion criteria and provided stool samples at all three time points (T1, T2, and T3). This complete-case analysis approach was determined *a priori* to ensure robust longitudinal comparisons and to avoid potential biases from imputing missing microbiota data.

**Table 1:** Baseline characteristics of study participants.

T1	Total	CON	RP + T	HP + T	*p*-value
Sociodemographic characteristics					
Participants (*n*)	112	39	35	38	0.890
Sex [*n* (f/m) (% females)]	58/54 (51.8%)	21/18 (53.8%)	18/17 (51.4%)	19/19 (50.0%)	0.886/0.946/0.943
Age (years)^Ø^	72.8 (4.7)	72.7 (4.7)	72.4 (4.4)	73.2 (5.0)	0.761
Clinical examination					
Carbohydrate intake^Δ^ (g/kg BW/d)	2.50 (1.45)	2.22 (1.76)	2.67 (1.37)	2.25 (1.20)	0.643
Fat intake^Δ^ (g/kg BW/d)	0.98 (0.65)	0.91 (0.51)	1.07 (0.60)	1.00 (0.62)	0.886
Protein intake^Δ^ (g/kg BW/d)	0.80 (0.40)	0.80 (0.40)	0.90 (0.30)	0.80 (0.50)	0.352
Energy intake^Δ^ (kcal/d)	1,727 (869)	1,700 (994)	1,925 (833)	1,666 (890)	0.341

^Ø^Data are expressed as mean ± SD. ^Δ^Data are expressed as median ± IQR. *p-values ≤ 0.05 show significant differences (chi-square test or ANOVA, respectively); CON, control group = observation only; RP + T, recommended protein group + resistance training; HP + T, high protein group + resistance training.

### 2.3 Sports and dietary intervention

A detailed study description has been published by our group (11). In summary, the protein intake of the participants was monitored, and HP + T group participants were provided with protein-rich foods that are readily available in stores, such as high-protein bread, puddings, dairy products, soups, and pea protein sticks. The RP + T group received foods with medium protein content, such as milk products, homemade muffins, and bars, to compensate for the energy intake by the HP + T group. A protein intake of 1.07 g/kg body weight/day for the RP + T group and 1.62 g/kg body weight/day for the HP + T group for 17 weeks based on an initial nutritional assessment (T1) was achieved. At week 9 (T2), guided progressive resistance training was initiated for 8 weeks (T3). After each training session, the participants of the groups received a supplement (HP + T protein supplement; RP + T an isocaloric carbohydrate alternative). The total caloric intake was adapted according to sex-specific recommendations based on D-A-CH reference values (12). CON group participants did not receive any supplements or resistance training and remained on their baseline protein intake throughout the study period. Anthropometric data, body composition, physical function, muscle quality, and selected blood parameters were recorded at T1, T2, and T3, respectively.

### 2.4 Stool sampling

Stool sampling kits containing labeled sterile containers with a spatula, thermal bag, feces catcher, and instructions were provided to the participants at T0. Stool samples were collected at three time points (T1, T2, and T3) in exchange for a new sampling kit. The samples were collected within 24 h and stored below 4 °C until delivery. Samples were aliquoted on the same day of collection, shock-frozen in liquid nitrogen, and stored at −80 °C until further analysis.

### 2.5 DNA extraction and multiplex-barcoding PCR

Microbial DNA was extracted from stool samples by automated purification using a QIAamp® Fast DNA Stool Mini Kit on a QIAcube. The procedure was performed according to the handbook Protocol for Pathogen detection (version March 2014). After suspension and lysis of 200 mg of the sample, 200 μL of the supernatant was used for the extraction. The concentration and purity of the purified DNA were checked using a NanoDrop 1000 system (Thermo Fisher Scientific Inc.) before storage at −20 °C.

The microbiota community composition was analyzed by amplification and sequencing of the V4 region of the bacterial and archaeal 16S rRNA genes at the Joint Microbiome Facility (Medical University of Vienna and University of Vienna; Project ID JMF-1901-3) using an Illumina MiSeq-based highly multiplexed gene amplicon sequencing approach (13) with 515F (GTGYCAGCMGCCGCGGTAA) and 806R (GGACTACNVGGGTWTCTAAT) as primers (14, 15). Sequencing and raw data processing were performed as described previously (13). Amplicon pools were extracted from raw sequencing data using the FASTQ workflow in BaseSpace (Illumina) with default parameters.

Demultiplexing was performed using the Python package demultiplex (Laros JFJ)^1^, allowing one mismatch for the barcodes and two mismatches for the linkers and primers.

Amplicon sequence variants (ASVs) were inferred using the DADA2 R package v1.12.33, applying the recommended workflow (16). FASTQ reads 1 and 2 were trimmed to 220/150 nt, with allowed expected errors of 2, respectively.

ASVs were classified according to the SSU rRNA gene sequences using SINA version 1.2.11, and the SILVA database SSU Ref NR 99 release 132 (16–19).

### 2.6 Monitoring protein absorption and digestion by residual stool energy

Protein absorption was monitored using a bomb-calorimetry-based method (20). Approximately 10 g of stool was transferred to a 50 mL falcon tube, and 10 mL of deionized water was added. The sample was homogenized using an Ultra-Turrax for approximately 10 s. After mixing, the residual sample on the Ultra-Turrax was homogenized with 5 mL of deionized water and added to the sample. The samples were stored in a sloping position to maximize their surfaces and frozen at −30 °C. The frozen samples were freeze-dried for approximately 60 h. After weighing, the samples were repeatedly freeze-dried for 2 h, until the change in weight was less than 1%. Dried samples were ground, and approximately 0.5 g was pressed into pellets for bomb calorimetry.

### 2.7 Monitoring GI inflammation by calprotectin ELISA

Calprotectin levels in stool samples were measured using a commercial ELISA Kit (21). Stool samples (100 mg) were aliquoted, extracted, and measured according to the kit manual for manual extraction using FLUOstar OPTIMA (22). The results were normalized to the weights of the samples.

### 2.8 Statistical analysis

Statistical analyses were performed using R version 4.2.1 (23) and RStudio version 2022.07.1 (24). Baseline data for the groups were expressed as mean ± SD or as median ± IQR according to the results of a Shapiro–Wilk test and tested for group differences using a chi-squared test or ANOVA.

**Figure 2:**
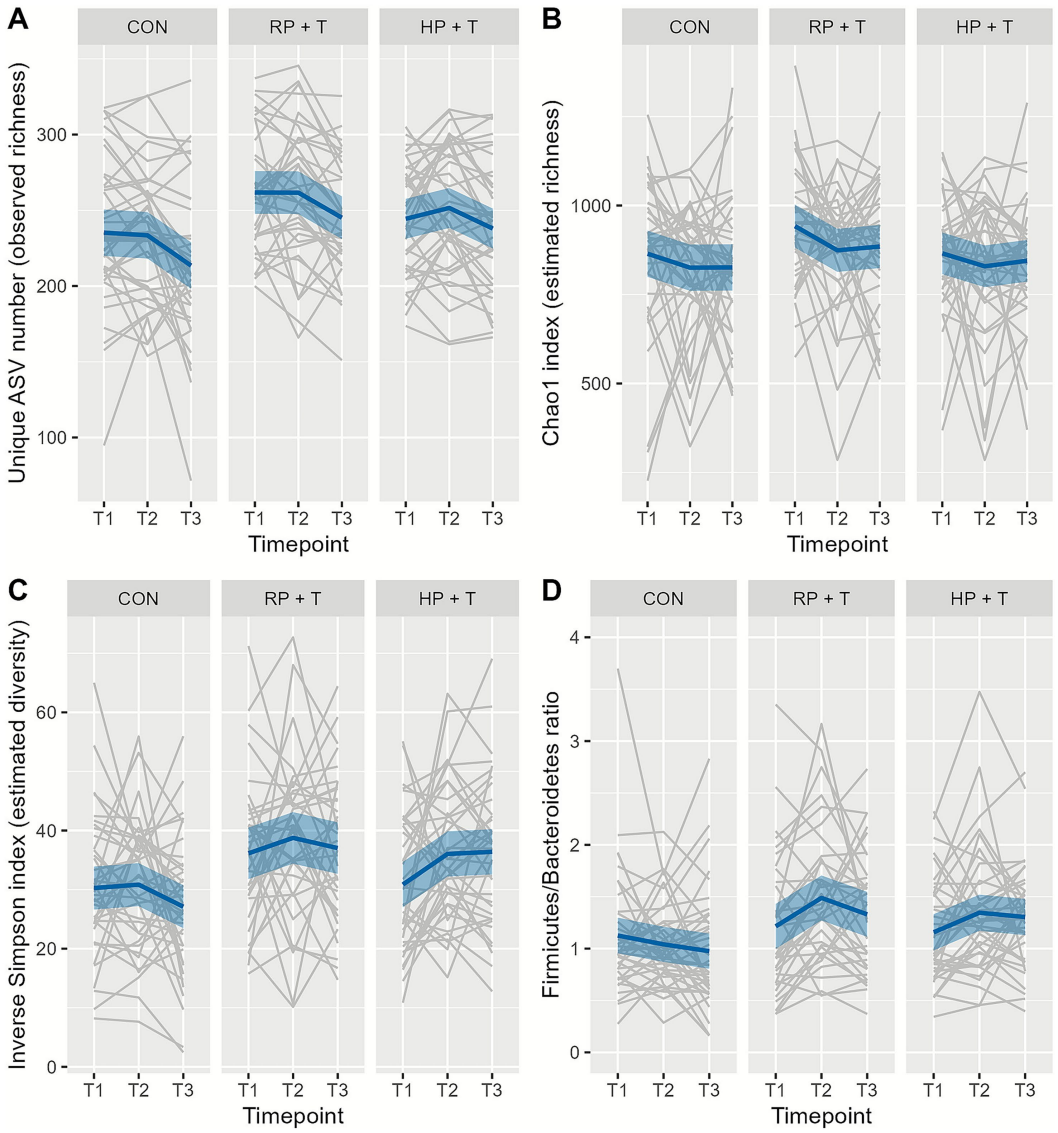
Changes in gastrointestinal microbiota **(A)** observed richness, **(B)** estimated richness, **(C)** estimated diversity, and **(D)** Firmicutes/Bacteroidetes ratio by intervention group (CON, control group = observation only; RP + T, recommended protein group + resistance training; HP + T, high protein group + resistance training) and timepoints (T1 represents baseline, T2 numbers after dietary intervention, and T3 after dietary and training intervention). The gray lines represent single participants, the dark blue lines represent the mean, and the light blue area represents the 95% CI. There were no significant changes within the groups throughout the time point tested using the repeated-measures linear mixed-effects model (*α* = 0.05); however, individual variation (gray lines) shows substantial fluctuation.

**Figure 3:**
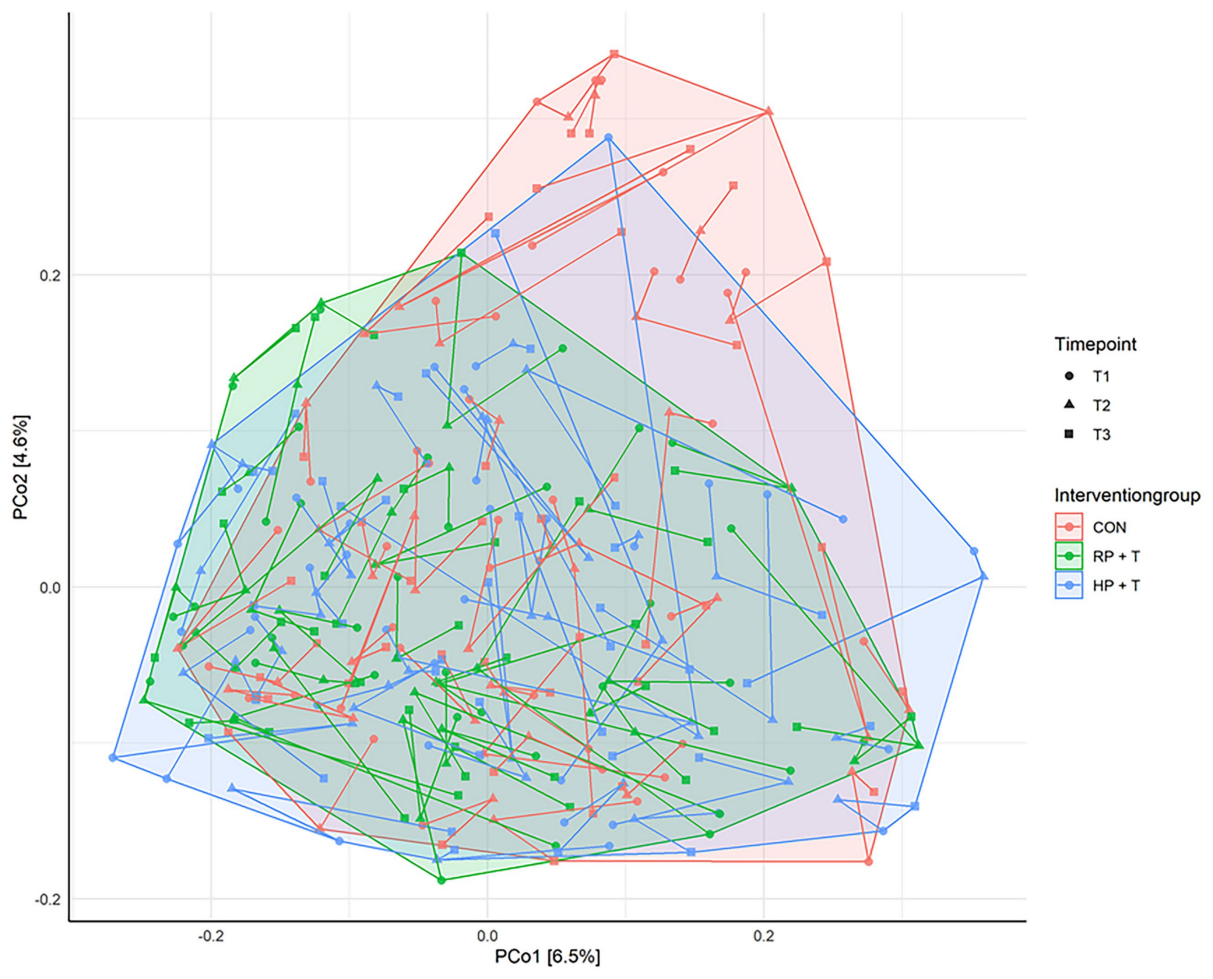
Principal coordinate (PCo) analysis with Bray–Curtis distance measure and untransformed species abundances was calculated using the ampvis2-package in R. The lines connect the three time points for each participant. Gastrointestinal microbiota-based ASVs below 0.1% in any stool sample were removed prior to analysis. The eigenvalues of PCo-Axis 1 and 2 are indicated as percentages in the axis titles. The groups showed complete overlap.

**Figure 4:**
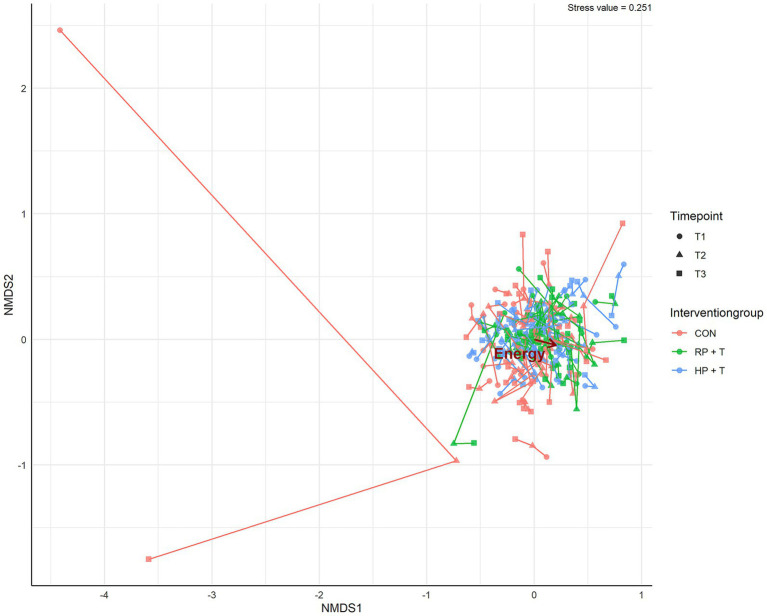
Non-metric multidimensional scaling analysis (nMDS) with the Bray–Curtis distance measure and untransformed species abundances was calculated using the ampvis2-package in R. The initial numerical factors used in the analysis were energy (kcal/day), carbohydrates (g/kg body weight/day), fat (g/kg body weight/day), protein (g/kg body weight/day), BMI (kg/m^2^), handgrip strength, maximum walking time 6 m, 6-min walking test, timed up and go test, age (years), and sex as nominal factors. Gastrointestinal microbiota-based ASVs with a relative abundance of less than 0.1% in all stool samples were removed. Goodness is shown in the upper-right corner as the stress value (not acceptable). A very short arrow length (energy) indicates a minimal correlation between the variable and ordination. The Mantel test revealed no significant correlation (*α* = 0.05). The groups showed complete overlap.

Changes in the GI microbiota composition were tested for the three groups across the three time-points each with repeated-measures linear mixed-effect model for observed richness, estimated richness, estimated diversity, and Firmicutes/Bacteroidetes ratio based on rarefied subsamples. The subsample size was defined as 2,250 sequences per sample, calculated by 95% size of the smallest sample size. For PCoA, nMDS, and PermANOVA, ASVs below 0.1% relative abundance in any sample were excluded. Details of the packages and settings are shown in Figures 2–4. A total of 3,299 individual ASVs were tested with a repeated-measures linear mixed-effect model with intervention group, time point, and their interaction as fixed effects, random intercept for participants (≡ repeated measures) and multiple testing correction using the statistical method of false-discovery rate (FDR; Benjamini–Hochberg procedure) at a level of *α* = 0.05.

For all tests, *α* = 0.05 was set as the threshold of statistical significance. The FDR was used to correct for multiple testing.

## 3 Results

### 3.1 Study population

Of the 136 participants aged 65 and 85 years at baseline, 116 completed the study, and 112 were included in the analyses of the GI microbiota (see Figure 1). Sex was equally distributed among the three groups, and baseline data did not differ, as shown in Table 1. Anthropometric data, body composition, and comorbidities have been evaluated and published elsewhere (11).

### 3.2 Nutritional intervention

Baseline intake of macronutrients was evaluated for the subgroup included in GI microbiota analysis (n = 112) and showed no group differences when compared to the total study group (*n* = 116). Throughout the study, carbohydrate and fat intakes did not change significantly. After T1, protein intake increased in the RP + T groups (T1-T2: *p* = 0.003; T1-T3: *p* = 0.005) and HP + T (T1-T2: *p* < 0.0001; T1-T3: p < 0.0001) as planned, while it remained constant over the study period in the CON group. Based on protein intake related to body weight, the groups reached a stable protein intake of approximately 0.83 ± 0.24 g/kg body weight in CON, 1.07 ± 0.25 g/kg in RP + T, and 1.62 ± 0.37 g/kg in HP + T until the end of the intervention.

### 3.3 Monitoring of GI microbiota composition

**Figure 5:**
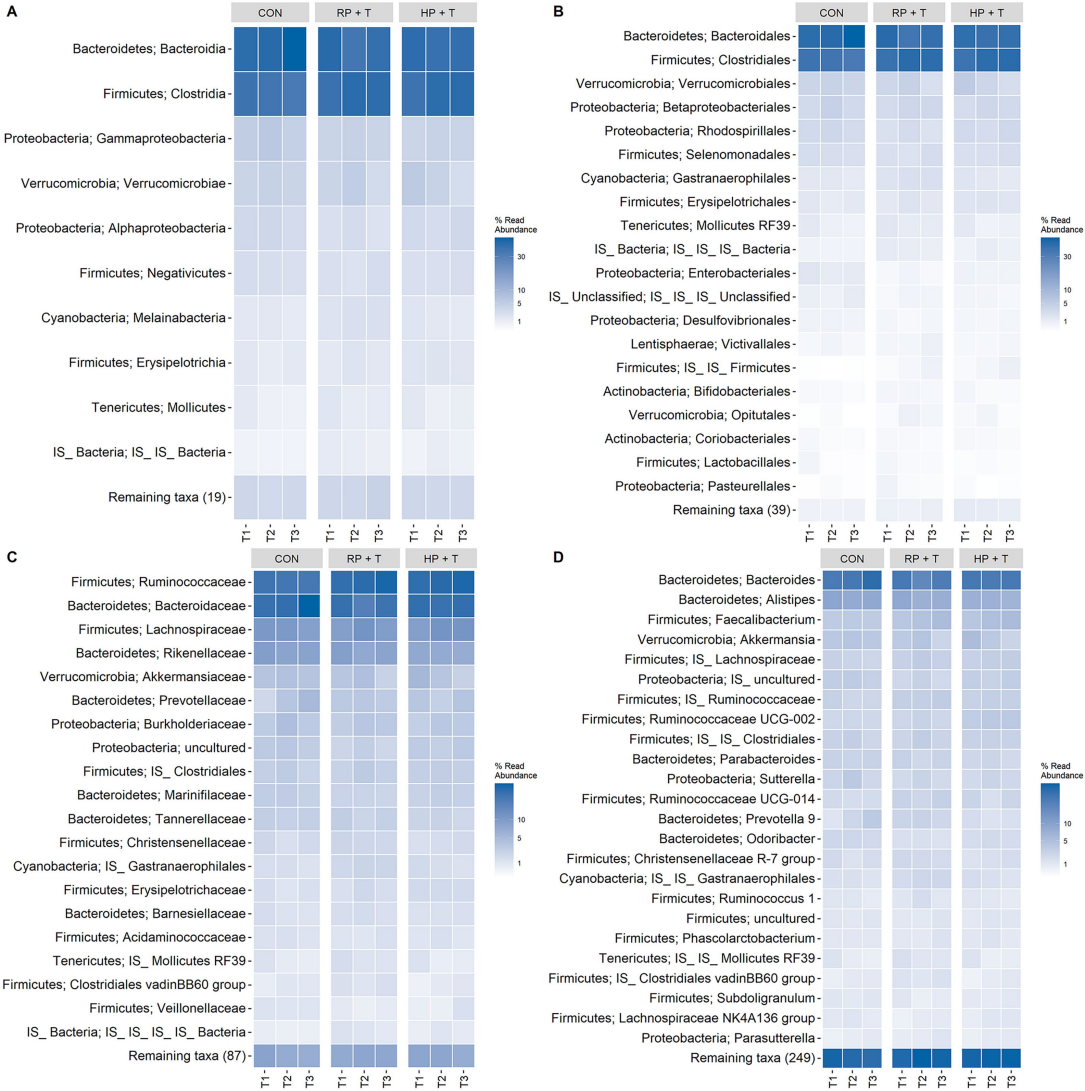
Heatmaps of relative ASV counts aggregated by **(A)** class level, **(B)** order level, **(C)** family level, and **(D)** genus level, grouped by time point, and facet by intervention group. A pairwise permANOVA (pairwise.adonis2) indicated no significant difference in the centroids of the time-point data within each intervention group. Therefore, it can be assumed that there were no differences or significant changes in the composition of gastrointestinal microbiota throughout the intervention within each intervention group.

One hundred and twelve participants provided stool samples at all three time points and met the inclusion criteria. 16S rRNA gene amplicon sequencing was used to analyze the composition of the GI microbiota. Interestingly, no significant changes were observed after intervention. As shown in Figure 5, microbiota richness, expressed as the number of unique amplicon sequence variants (ASV) on a rarefied depth dataset (*n* = 2,250 reads/sample), and markers of bacterial diversity did not change (tested using repeated-measures linear mixed-effects models with FDR correction, *α* = 0.05).

Tests for changes in beta diversity in and between the microbiota communities (principal coordinates analysis) of the study groups showed no significant longitudinal shifts or clustering by subgroups (see Figure 2).

Non-metric multidimensional scaling analysis was used to evaluate the influence of single factors on microbiota. Based on the study design, we focused on the nutritional influence and impact of the strength training phase. The details are presented in Table 1. Only total energy intake was linked to shifts in the microbiota, but this correlation was not significant, as shown in Figure 3.

Based on this finding and with permANOVA testing of the counts of ASVs (see Figure 4), we did not observe shifts in the microbiota of the older adults by the intervention of increased protein intake.

### 3.4 Monitoring absorption and tolerance of increased protein intake

Protein absorption and tolerance were assessed using residual energy measurements and inflammatory markers in stool samples across all time points (T1, T2, and T3).

Bomb calorimetry analysis of freeze-dried stool samples showed no significant changes in residual energy between the baseline and the end of the intervention across all groups.

Calprotectin, a marker of GI inflammation, remained stable throughout the intervention period.

Statistical analysis using linear mixed-effects models confirmed that there were no significant changes in residual energy or inflammatory markers in the intervention groups compared to baseline.

These findings suggest that increased protein intake up to 1.62 ± 0.37 g/kg body weight was well-tolerated by the participants.

The data are summarized in the Supplementary material (Supplementary Figures 1, 2 and Supplementary Tables 1, 2).

## 4 Discussion

Human diet shapes the GI microbiota and influences overall health and aging. Diets rich in fiber, plant-based foods, fermented products, and polyphenols promote microbiota diversity and reduce chronic disease risk (25). Conversely, diets high in fat, sugar, processed foods, and red meat disrupt GI balance, thereby increasing dysbiosis and health risks (26).

For older adults, age-related GI microbiome changes—such as reduced diversity and beneficial bacteria—are linked to frailty and chronic diseases (2). A balanced, fiber-rich diet with diverse protein sources is crucial for maintaining GI health and supporting healthy aging (27).

In particular, adequate protein intake is considered crucial for maintaining or enhancing muscle quality and mass, thereby helping to prevent sarcopenia and its associated adverse health outcomes—especially when combined with resistance training (28, 29). However, a high dietary protein intake may exert adverse effects, particularly in older adults with compromised gastrointestinal efficiency or declining renal function (30). Therefore, we aimed to investigate the effects of 17 weeks of moderate and high protein intake, with and without strength training, on the GI microbiota of community-dwelling older adults.

We were successful in achieving a personalized protein intake of 1.07 ± 0.25 g/kg BW in the RP + T group, which is very close to the recommendation in the D-A-CH region, and we doubled the baseline protein intake to 1.62 ± 0.37 g/kg body weight in the HP + T group and followed this regimen for 17 weeks without a significant increase in carbohydrate and fat intake. One innovation of this study was that protein intake was not administered as a protein powder or supplementation, as in many other studies but was provided with commercially available food products of animal and plant origin, either rich in protein for the HP + T group (e.g., protein-rich milk products, bars, puddings, protein-rich bread, bacon crisps, protein-rich soups, pea protein sticks, and recipes for self-prepared foods) or similar foods with regular protein content for the RP + T group (e.g., milk products, bars, bread, soups, and self-made vegetable muffins). In total, we provided more than ten tons of food to the participants. Individual protein intake was recorded daily by the respective participants and monitored throughout the entire study period with nine 24-h recalls per participant.

Following this dietary modification, the protein content in the HP + T group increased from 14% at baseline to approximately 25% of total energy intake. Stool samples were collected at three time points throughout the study to monitor potential changes in GI microbiota composition, residual energy in stool, and the inflammation marker calprotectin.

However, it may also promote proteolytic fermentation, resulting in the formation of putrefactive metabolites (31, 32). Jantchou et al. (2010) demonstrated that a high-protein intake, especially from animal sources, is associated with an increased risk of inflammatory bowel disease (IBD) (33).

Stool microbiota diversity and composition were analyzed using 16S rRNA gene amplicon sequencing. Despite the dietary changes in protein intake, alpha and beta diversities did not change throughout the study in either intervention group. Figure 5 shows the development of observed richness, estimated richness, estimated diversity, and the Firmicutes/Bacteroidetes ratio. Furthermore, a principal coordinate analysis (see Figure 3) and a non-metric multidimensional scaling analysis (see Figure 4) showed a complete overlap between the groups and only fluctuations within individual participants.

To evaluate potential shifts at a finer taxonomic resolution, we examined the relative abundance of bacterial genera that are known to be associated with protein metabolism and health outcomes in older adults. The key genera examined included *Bifidobacterium* and *Lactobacillus* (beneficial bacteria that decline with age), *Akkermansia* (mucin-degrading bacteria associated with metabolic health), butyrate-producing genera (*Faecalibacterium*, *Roseburia*), and genera associated with protein fermentation. The repeated-measures linear mixed-effects model did not show significant changes in any of these measures. A pairwise permANOVA on relative ASV counts aggregated by different phyla (see Figure 5) showed sustained abundance of bacterial phyla. No statistically significant changes were observed in the relative abundance of these key genera across the time points or between the intervention groups.

Although our study confirms the structural stability of the GI microbiota, it should be noted that the structural composition alone does not reflect full functionality. High protein intake can increase proteolytic fermentation and potentially lead to putrid metabolites, such as phenols, indoles, sulfides, ammonia, amines, and trimethylamine N-oxide (TMAO) precursors. These metabolites may influence host health independently of compositional shifts. The observed stability in the microbiota composition indicated that increased protein intake did not disrupt the overall community structure.

This was an unexpected but very positive observation, proving that a shift to a high-protein diet via conventional food products over more than 4 months did not lead to negative outcomes in the GI microbiota of older adults.

Doubling protein intake through protein-rich foods, in alignment with current dietary recommendations, does not appear to adversely affect GI microbiota composition when predominantly provided via whole food sources. Adequate protein intake is the basis for preventing sarcopenia; however, only if the increased amount has no adverse effects on other health indicators such as GI health (34).

Similarly, residual energy and levels of the inflammatory marker fecal calprotectin did not differ significantly between or within the groups. There was no evidence of an increase in either of these markers. Residual energy can be used as a marker for digestion. An increase in residual energy based on the increased protein intake, which could be a sign of undigested protein reaching and passing through the large intestine (35), was not observed.

Taken together with the observed stability in GI microbiota diversity and composition, these findings support the feasibility of increasing protein intake in older adults to at least 1.6 g/kg body weight—a level shown to be effective in preserving and enhancing muscle mass with advancing age (36). This is needed, for example, to improve muscle protein synthesis or hand grip strength.

There is animal data that supplements our findings. The source of proteins and their quality appear to be a major driver of microbiota shifts and health outcomes, especially meat-based vs. non-meat-based proteins. Plant-based proteins, primarily soy and legume proteins, as well as dairy proteins such as casein, tend to be less obesogenic by affecting appetite, altering nitric oxide production, and have a high content of branched chain amino acids (BCAAs), and are more beneficial for microbiota balance (e.g., increased abundance of *Akkermansia* and *Bifidobacterium*, fewer sources of TMAO-metabolites) (37).

The application form is also important; whole-food diets with an increased number of unprocessed foods, as it was the focus of our study design, could mask macronutrient-specific effects. However, studies employing isolated macronutrients consistently demonstrate that high-fat, carbohydrate, and fiber content exert a more pronounced influence on microbiota composition than isocaloric high-protein diets (38–41).

First, the older adult microbiome exhibits inherent resilience and reduced plasticity compared to younger cohorts, as documented in aging research (42–44). Second, our whole-food intervention approach, which provides protein through diverse food matrices rather than isolated supplements, may have buffered macronutrient-specific effects. The complexity of whole foods, containing fiber, polyphenols, and other bioactive compounds, could mask direct protein effects on microbiota (45). Third, the food-based format ensured adequate co-ingestion of dietary fiber and other nutrients that may counteract potential negative effects of increased protein fermentation (46). Fourth, resistance training itself may have beneficial effects on GI health through improved intestinal transit time and systemic metabolic changes (47).

Fiber intake was assessed at all three time points to evaluate its potential role as a confounding variable. The average daily fiber intake remained stable in all groups throughout the study. However, due to missing product data for dietary fiber, the data were not fully sufficient to perform more detailed analyses and correlations in this subanalysis. To address this major limitation, the Healthy Eating Index by EPIC was calculated in a supplementary subanalysis (48). This showed that the increased intake of protein products had no particular influence on dietary quality.

The strength of our study is the two-phase study design, with an initial nutritional phase and a subsequent combined nutritional and exercise intervention, which has not been performed before in an older adult cohort. Furthermore, we achieved doubling of the baseline protein intake with a food-based approach and not by supplementation, which is an important aspect of dietary adherence, specifically in this age group. We individualized and tightly controlled the participants’ diet by performing 24-h recalls every 7–10 days; therefore, we believe that we kept the reporting error at a minimum. As the intensity of the strength training program was controlled by subjective experience (RPE scale), the resulting intensity was moderate, which we believe resulted in a low dropout rate during the strength training intervention. Microbiota analysis was performed in the stool samples and considered both nutritional and combined nutritional and training interventions, all of which potentially could have affected the GI microbiota. Our study was conducted exclusively on community-dwelling older adults (65–85 years) in Vienna, Austria, representing a relatively homogeneous Central European population. This limits the generalizability of our findings to populations with different genetic backgrounds, dietary traditions, baseline microbiota compositions, and environmental exposure. Future multicenter studies encompassing diverse geographic and ethnic populations would be valuable to confirm the generalizability of these findings. Another limitation is that we did not measure trimethylamine-N-oxide (TMAO) in this study, which is also currently under evaluation as a possible metabolic marker for cardiovascular risks.

## 5 Conclusion

A habitual diet with recommended or high protein intake, with or without strength training for over 17 weeks, showed no detectable adverse effects on the GI microbiota composition of 112 community-dwelling adults aged 65–85 years. Despite the increased protein intake of up to 1.62 g/kg body weight, GI microbiota richness, diversity, and composition, no significant changes within or between groups were observed. Residual energy and inflammatory markers indicated that a higher protein intake was well tolerated.

## Data Availability

The datasets presented in this study can be found in online repositories. The names of the repository/repositories and accession number(s) can be found at: https://www.ncbi.nlm.nih.gov/bioproject/PRJNA1275818/.

## Footnotes

1 github.com/jfjlaros/demultiplex.
