# Supplementary material for: The impact of a high-protein diet with strength training on the gastrointestinal microbiota in community-dwelling older adults: subanalysis of a randomized controlled trial
Source: Front Nutr. 2026 Jan 20;12:1712451. doi: 10.3389/fnut.2025.1712451 (PMC12864401; doi:10.3389/fnut.2025.1712451)
Supplement: Supplementary file 1 [file Data_Sheet_1.docx]

Supplementary Material


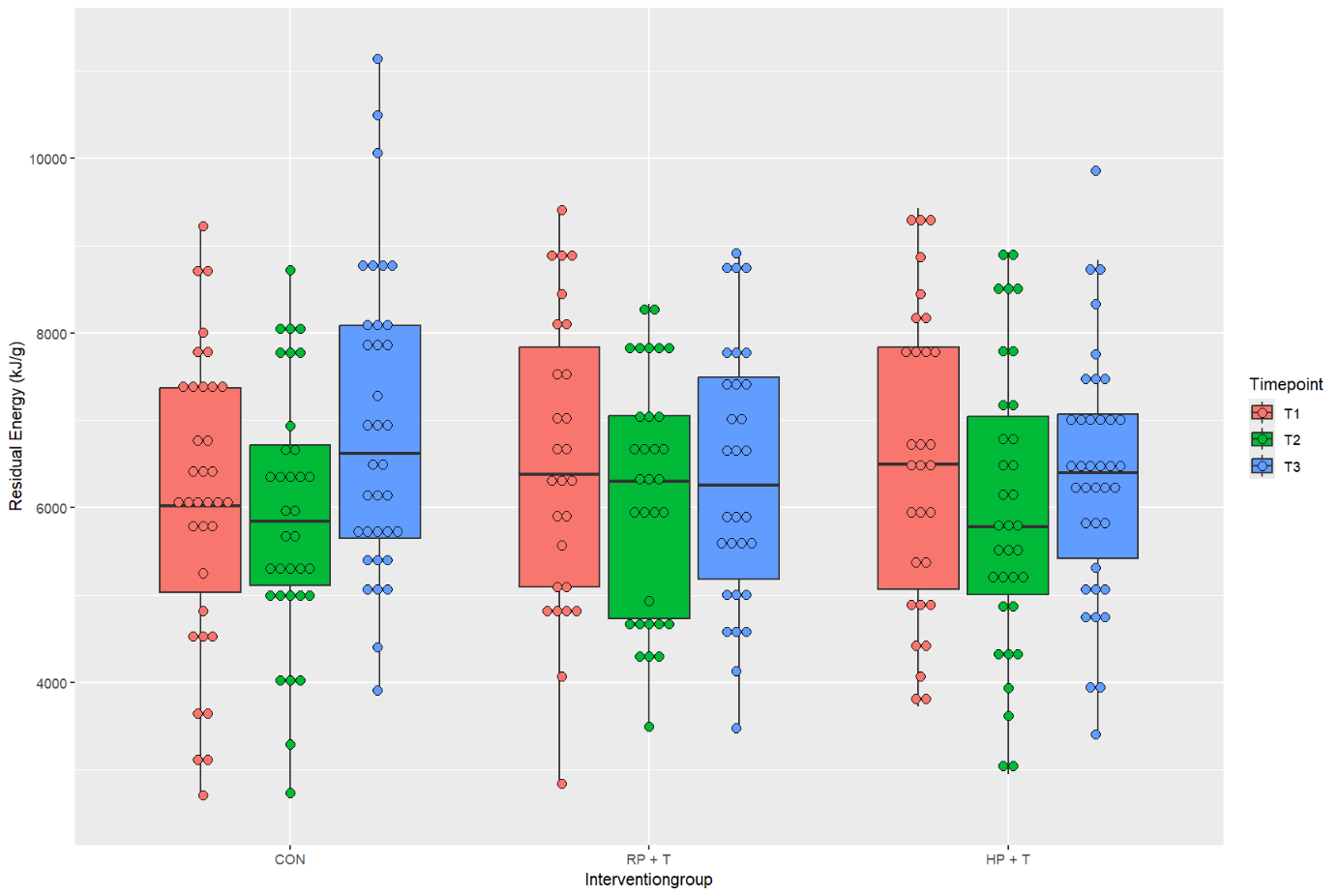


**Supplementary Figure 1.** The boxplot shows residual energy [kJ/g] of human stool across three intervention groups and timepoints.

Medians and spreads are similar, with values mostly between 5,000 and 7,000 kJ/g. High variability and numerous outliers are present, but no clear effect of intervention or timepoint is evident in the data.

(CON; control group = observation only; RP + T; recommended protein group + resistance training; HP + T; high protein group + resistance training); and Timepoints (T1 represents baseline, T2 numbers after dietary intervention and T3 after dietary and training intervention).


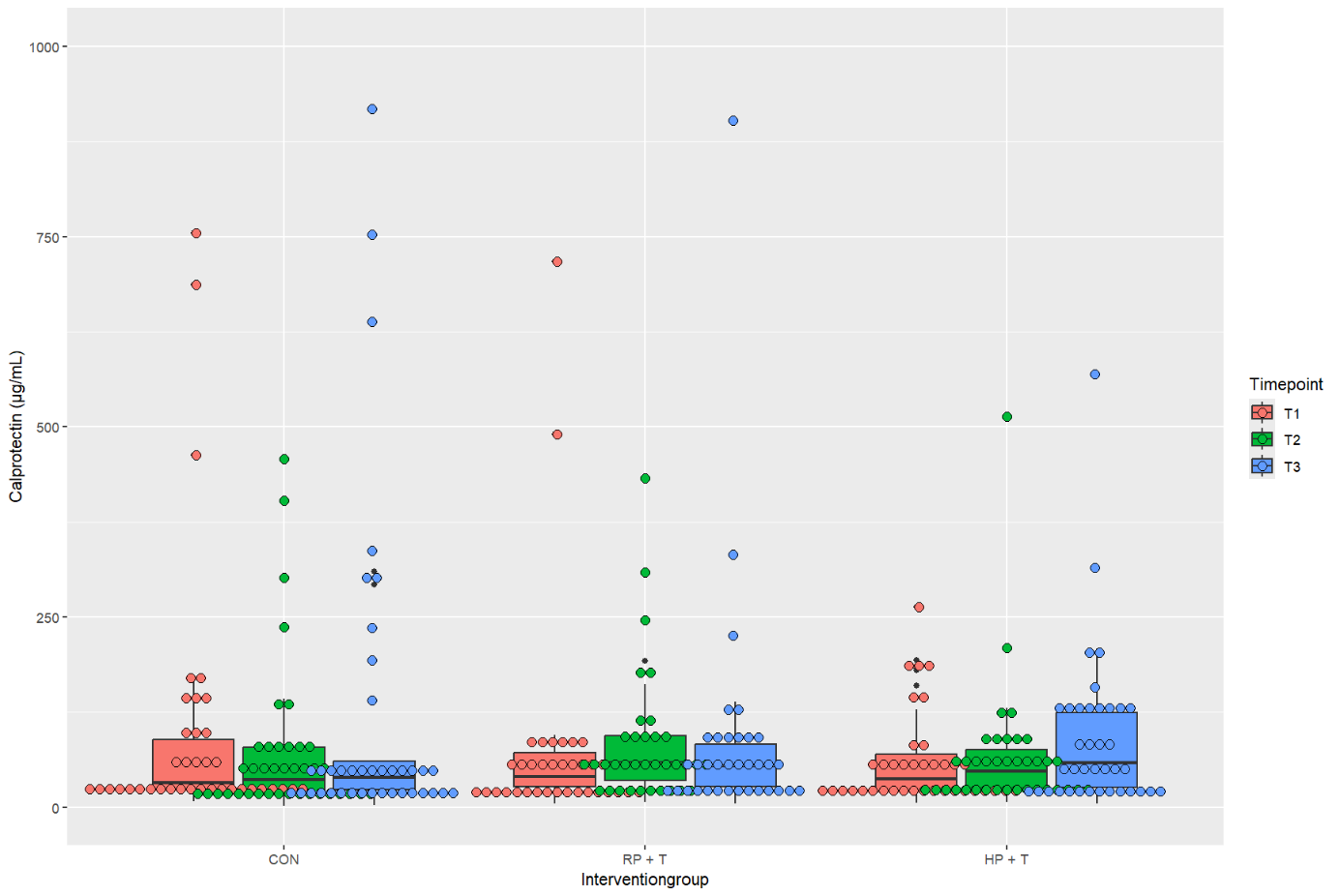


**Supplementary Figure 2.** Boxplot of the ELISA-measured levels of fecal calprotectin in [µg/mL].

The boxplot shows calprotectin levels across three intervention groups and timepoints. Most values cluster below 200 µg/mL, but each group displays high outliers. 2 values were masked in boxplot (CON T1: 1,870.9 µg/mL and HP + T T2 2,290.7 µg/mL). Medians and spreads are similar throughout, indicating no clear effect of intervention or time.

(CON; control group = observation only; RP + T; recommended protein group + resistance training; HP + T; high protein group + resistance training); and Timepoints (T1 represents baseline, T2 numbers after dietary intervention and T3 after dietary and training intervention).

**Supplementary Table 1.** Longitudinal data of Residual Energy of stool of the study participants

| Residual Energy [kJ/g] | T1 | T2 | T3 | p-value |
| --- | --- | --- | --- | --- |
| CON ^Ø^ | 6106 (1662) | 5919 (1453) | 6932 (1743) | 0.3557 |
| RP + T ^Ø^ | 6503 (1685) | 6168 (1392) | 6372 (1508) | 0.8308 |
| HP + T^Ø^ | 6540 (1720) | 5980 (1641) | 6354 (1390) | 0.3444 |
| ^Ø^Data are expressed as mean value ± SD. *p-values ≤0.05 show significant differences (ANOVA of Fit Linear Mixed-Effects Models, including age as a covariate); CON (control group = observation only); RP + T (recommended protein group + resistance training); HP + T (high protein group + resistance training); | | | | |

**Supplementary Table 2.** Longitudinal data of fecal calprotectin levels of the study participants

| Fecal Calprotectin [µg/mL] | T1 | T2 | T3 | p-value | |
| --- | --- | --- | --- | --- | --- |
| CON ^Ø^ | 145.0 (329.0) | 75.2 (103.0) | 122.0 (210.0) | 0.1008 | |
| RP + T ^Ø^ | 76.8 (137.0) | 85.2 (88.9) | 90.4 (155.0) | 0.5923 | |
| HP + T^Ø^ | 60.6 (60.9) | 123.0 (371.0) | 91.3 (104.0) | 0.4578 | |
| ^Ø^non-negative Data are expressed as mean value ± SD. *p-values ≤0.05 show significant differences (ANOVA of Fit Linear Mixed-Effects Models); CON (control group = observation only); RP + T (recommended protein group + resistance training); HP + T (high protein group + resistance training); | | | | |  |
